# Burden and factors associated with depression symptoms among older adults in Madinah, Saudi Arabia

**DOI:** 10.3389/fpubh.2026.1824647

**Published:** 2026-05-14

**Authors:** Muayad Albadrani

**Affiliations:** 1 Department of Family and Community Medicine and Medical Education, College of Medicine, Taibah University, Madinah, Saudi Arabia; 2 Health and Life Research Center, Taibah University, Madinah, Saudi Arabia

**Keywords:** associated factors, depression, GDS-15, LSNS-6, mental health, older adults

## Abstract

**Background:**

Depression is one of the most significant contributors to disability across the world, and it is particularly prevalent in older people due to a variety of factors, including chronic illness, loss of function, and social isolation. The general population in Saudi Arabia is getting progressively older; however, data on geriatric depression are scarce by region. This study aimed to assess the burden and factors associated with depression symptoms among older adult residents in Madinah.

**Methodology:**

This is a cross-sectional study conducted among geriatric patients in Madinah, Saudi Arabia, from June 1 to August 31, 2025. Information was obtained from respondents through interviews. Statistical significance was considered when the *P* value was less than 0.05. Due to the cross-sectional design, causal relationships cannot be inferred.

**Results:**

A total of 322 older adults were enrolled; most were male, married, and aged 60–70 years. The prevalence of depressive symptoms (GDS-15 ≥5) was 52.5% (95% CI: 47.0%−57.9%). In bivariate analyses, higher depression scores were significantly associated with older age, female sex, widowed status, lower education and income, living alone, poorer self-rated health, physical inactivity, ADL difficulties, polypharmacy, and the presence of chronic diseases (all *p* < 0.05). Multivariable analysis identified unemployment (OR = 7.66), having no chronic disease (OR = 10.62), taking no medications (OR = 3.93), poor self-rated health (OR = 0.18 for excellent health), and physical inactivity (OR = 0.15 for daily exercise) as independent predictors of depression. Lower LSNS-6 scores (greater social isolation) were also significantly associated with older age, female sex, living alone, selected chronic conditions, polypharmacy, poorer self-rated health, lower physical activity, and greater functional limitation (all *p* < 0.05).

**Conclusion:**

Depression is a significant health problem affecting more than half of older adults in Madinah. Unemployment, physical inactivity, poor self-rated health, and having no chronic disease are independent correlates. Widowed status, female sex, older age, and low socioeconomic status were also associated in bivariate analyses. These findings underscore the need for early detection, integrated care, and culturally tailored interventions to promote mental health and quality of life in Saudi Arabia's aging population.

## Introduction

Aging is an irreversible biological process of unique dynamics. Its meaning varies among cultures and is often viewed as a gradual decline of bodily functions that impact survival and reproductive performance. Such knowledge, therefore, affects our understanding of health and life in various societal contexts (1). Healthy aging was considered to be living independently without assistance in daily activities at an old age. Successful aging means that the older adults can perform their daily tasks independently. It also says that they can maintain their physical health, mental well being, and social connections (2). In developed societies, chronological age is widely considered an indicator of the onset of aging, and retirement commonly occurs between the ages of 60 and 65, signifying institutional old age. In some parts of the world, particularly in developed countries, old age is a condition where no amount is too little (3).

In old age, patients' depression often coexists. Studies suggest that almost 90% of people with present with symptoms of depression (4). When these diseases occur, the symptoms are usually exacerbated and prolonged (5). This negatively impacts the quality of life and is correlated with many diseases, including an increased mortality rate (6). A few studies emphasize the necessity of separating adaptive from pathological symptoms in old age, as such symptoms can often be interpreted as an immediate response to aging (7).

If left untreated, the severity of medical and psychiatric illness may be greater (8). If an appropriate diagnosis is not made, for example, subthreshold but hidden symptoms (symptoms not expressed), the help and treatment that such older adults people need may remain out of reach (9) Co-occurring pathological disorders may be associated with higher levels of psychological, physical, and social impairment than adaptive emotions or single conditions, as well as requiring more resources to manage and being linked with increased suicide risk (10).

Recently, there has been an increase in the worldwide older adults population. Life expectancy for the general population rose from 66.4 years in 2000 to 73.4 years in 2019 (11). In 2020, there were one billion older people in the world, with that number projected to double by 2050 (12). This increase may be a result of a better quality of life in the present day. In Saudi Arabia, according to recent estimates, the older adults population accounts for approximately 5% of the total population (13). Consistent with Vision 2030, the Kingdom is focused on improving the lives of its citizens in Saudi Arabia. Moreover, the vision aims to increase the average age at death to 80 by the end of 2030 (14).

A study from Saudi Arabia 30 years ago demonstrated that depression was present in 39% of older adults participants (15). The Saudi National Mental Health Survey (SNMHS) recently reported that major depressive disorder is the third most common mental health diagnosis in the kingdom and has been diagnosed over the lifetime in 3% of males and 9% of females (16). In addition, no studies have previously been conducted on depression among the older adults in Madinah (17). Madinah's unique socio-religious environment, emphasizing togetherness and spirituality, may be associated with protective effects against depression. Yet, rapid modernization, a lack of equity in accessing healthcare, and an influx of a transient population can be potential risks. This underscores the importance of local studies that can inform tailored interventions, policies, and resource allocation in the context of Saudi Arabia's Vision 2030 (18).

We aim to identify this gap by investigating the burden of depressive symptoms in older adults and the factors associated with them, using an in-depth cross-sectional study to identify significant correlations. Based on validated screening tools, this study aims to examine how sociodemographic and health-related factors contribute to depression in older adults. Furthermore, we explore the association between chronic diseases and depressive symptoms in this population. Depression (measured by GDS-15) is the primary outcome of this study. We hypothesize that lower social support, poorer health, and lower physical activity are associated with higher levels of depression.

## Methodology

*Study design and duration:* This study employed a cross-sectional observational design to provide the first population-based baseline estimate of geriatric depression in Madinah. Participants have consented to be re-contacted annually, and preparations are underway for a prospective cohort follow-up to assess incidence, temporality, and causal pathways, including social isolation, depression, and health-care utilization.

This study is reported in accordance with the Strengthening the Reporting of Observational Studies in Epidemiology (STROBE) guidelines for cross-sectional studies

*Study population:* Participants aged 60 years and older residing in Madinah. assessed through orientation and communication ability during the interview by trained data collectors, were excluded, which would prevent participation. Participants below 60 years or who have lived outside Madinah.

*Sample size:* The sample size was determined, assuming a 30% prevalence of depression among older adults, with 95% confidence and a 5% margin of error. At least 322 participants were enrolled. The final sample size met the minimum requirement, and no oversampling was done due to logistical and resource limitations.

*Sampling technique*: Participants were selected using a multistage random sampling method. First, Madinah was divided into urban and rural areas. Second, neighborhoods were randomly selected from each area. Third, households within selected neighborhoods were randomly sampled. Fourth, within each household, one eligible older adult (aged ≥60 years, a resident of Madinah, with severe cognitive impairment) was informally assessed during the interview, primarily based on how well participants could understand and respond to simple questions. If someone couldn't follow basic conversation or communicate clearly, they were excluded from the study. No formal or validated cognitive screening tool was used in this process.

The sampling frame comprised all households in the selected neighborhoods; a complete registry of all older adults residents was not available. Representativeness was assessed by comparing the age and sex distribution of the sample to published Madinah census data (General Authority for Statistics, 2025), which showed no major discrepancies.

*Data collection:* Data was collected in person during face-to-face interviews with participants at the community centers or clinics, or in their homes in Madinah, by trained data collectors to avoid leading questions and reduce interviewer and recall bias. The information obtained comprised Demographics and Health Information, covering age, sex, marital status, education, occupation, Health conditions, including chronic diseases, and medication use were documented. During the interview, participants self-reported their chronic diseases, medication use, self-rated health, physical activity, and difficulties with activities of daily living (ADL).

The Geriatric Depression Scale (GDS-15) in Arabic was applied. It is designed for older adults, with a score range of 0–15; a score of 5 or more is indicative of depression. The Arabic GDS-15 has been previously validated and shows good internal consistency [Cronbach's α = 0.83; Chaaya et al. (19)]. Social isolation was quantified using a validated 6-item Lubben Social Network Scale (LSNS-6) questionnaire. The Arabic LSNS-6 has also been validated, with robust internal consistency [Cronbach's α = 0.83; Trabelsi et al. (20)].

*Data entry and analysis:* The data were revised, cleaned, and extracted into an Excel sheet. Statistical analysis was conducted using SPSS (Statistical Package for the Social Sciences), version 26. Descriptive statistics were used to summarize the data. Categorical variables were reported as frequencies and valid percentages. In contrast, numerical variables were expressed as mean ± standard deviation (SD) for normally distributed data and as median with interquartile range (IQR), minimum, and maximum for non-normally distributed data. Normality was assessed using the Shapiro-Wilk test. GDS-15 was analyzed as both a continuous score and a categorical variable (≥5), while LSNS-6 was treated as a continuous variable. All other variables were treated as categorical. Data that were not normally distributed were described using the median (IQR).

The prevalence of depressive symptoms was calculated using the GDS-15 cutoff of ≥5, and its 95% confidence interval (CI) was computed using the Wilson score method. For bivariate comparisons, the Mann-Whitney U test was used for two-group comparisons and the Kruskal-Wallis test for comparisons involving more than two groups.

To identify independent predictors, multivariable binary logistic regression was performed with depression status (GDS-15 ≥5) as the dependent variable. All variables that were significant at *p* < 0.05 in bivariate analyses were entered simultaneously. Results are reported as adjusted odds ratios (aOR) with 95% CI and *p*-values. For social connectedness, multivariable linear regression was performed with the LSNS-6 total score as the continuous dependent variable, using the same set of independent variables. Results are reported as unstandardized regression coefficients (B) with 95% CI and *p*-values. Multicollinearity was assessed using variance inflation factors (VIF).

All questionnaires were reviewed for completeness immediately after each interview. No missing data were present for any variable in the final analytic sample (*N* = 322). Hence, no imputation or missing data handling was required

Statistical significance was considered when the *P* value was less than 0.05. To minimize bias, interviewers were trained to ask questions in a standardized and neutral manner, and interviews were conducted in private settings.

*Ethical considerations:* Ethical approval was obtained. All study procedures were conducted in accordance with applicable ethical guidelines. Participants' data were collected anonymously and treated with strict confidentiality.

## Results

**Figure 1:**
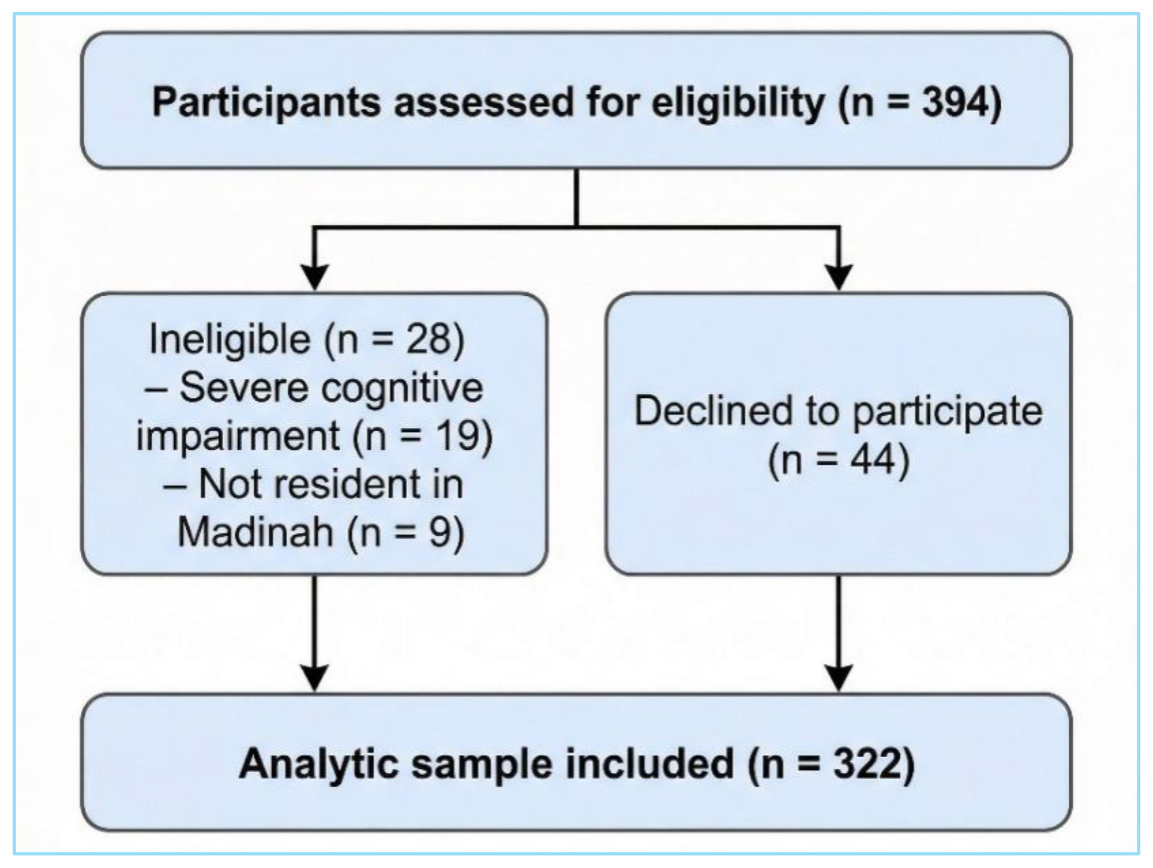
Participant flow diagram illustrating recruitment and inclusion of study participants.

Among the 322 older adults inhabitants of Madinah enrolled in the study, the overall response rate was 82%. The number approached (*n* = 394), ineligible (*n* = 28), refusals (*n* = 44), and the final analytic sample (*n* = 322) are detailed in Figure 1.

It can be concluded that there was a predominance of males (61.8%), and a considerable proportion fell within the age range of 60–70 years (74.1%). Most of them are married (72.0%) and living together with family members (65.8%). Educational status was diverse, with 24.2% being illiterate and 32.6% holding a bachelor's degree. The majority were retirees (66.5%) and reported a monthly income of more than 10,000 SAR (38.8%). Chronic diseases, particularly diabetes (69.6%) and hypertension (47.2%), were prevalent; 58.1% took three or more prescription medications daily. Nevertheless, 37.3% rated their health as very good, while few were engaged in physical activity, and only 17 % exercised at least three times a week. Functional independence appeared to be quite high, as 60.2% of patients reported being able to manage their daily activities, as indicated in Table 1.

**Table 1:** Socio-demographic for all older adults in Madinah, Saudi Arabia (*N* = 322).

Factor	Category	Number	Percentage
Age group (years) (*N* = 320)	60–70	237	74.1
	71–80	64	20.0
	81–90	19	5.9
Gender	Female	123	38.2
	Male	199	61.8
Marital status	Single or separate	8	2.5
	Married	232	72.0
	Divorced	12	3.7
	Widowed	70	21.7
Education level	Illiterate	78	24.2
	Primary to secondary school	121	37.6
	Bachelor's degree	105	32.6
	Postgraduate	18	5.6
Employment status	Unemployed	93	28.9
	Employee	15	4.7
	Retired	214	66.5
Monthly income (SAR)	< 3,000	74	23.0
	3,000–5,999	46	14.3
	6,000–9,999	77	23.9
	>10,000	125	38.8
Living arrangement	Live with their families	212	65.8
	Lives with other family members	29	9.0
	Living with children	38	11.8
	Living alone	43	13.4
Chronic diseases^*^	Diabetes	224	69.6
	Hypertension	152	47.2
	Arthritis	72	22.4
	Respiratory diseases	36	11.2
	Heart disease	55	17.1
	Other	33	10.2
	Nothing	26	8.1
How many prescription medications do you take daily?	None	57	17.7
	1–2	78	24.2
	3 or more	187	58.1
How would you rate your overall health?	Excellent	69	21.4
	Very good	120	37.3
	Good	96	29.8
	Fair to poor	37	11.5
How often do you exercise or engage in physical activity?	Daily	25	7.8
	3–5 times a week	30	9.3
	1–2 times a week	70	21.7
	Rarely	131	40.7
	Never	66	20.5
Do you have any difficulties with activities of daily living?	No difficulties	194	60.2
	Some difficulties	89	27.6
	Many difficulties	32	9.9
	Unable to perform without help	7	2.2

^*^Chronic-diseases item permitted multiple responses; therefore, column percentages could exceed 100%. ^*^ SAR = Saudi Arabian Riyal.

**Figure 2:**
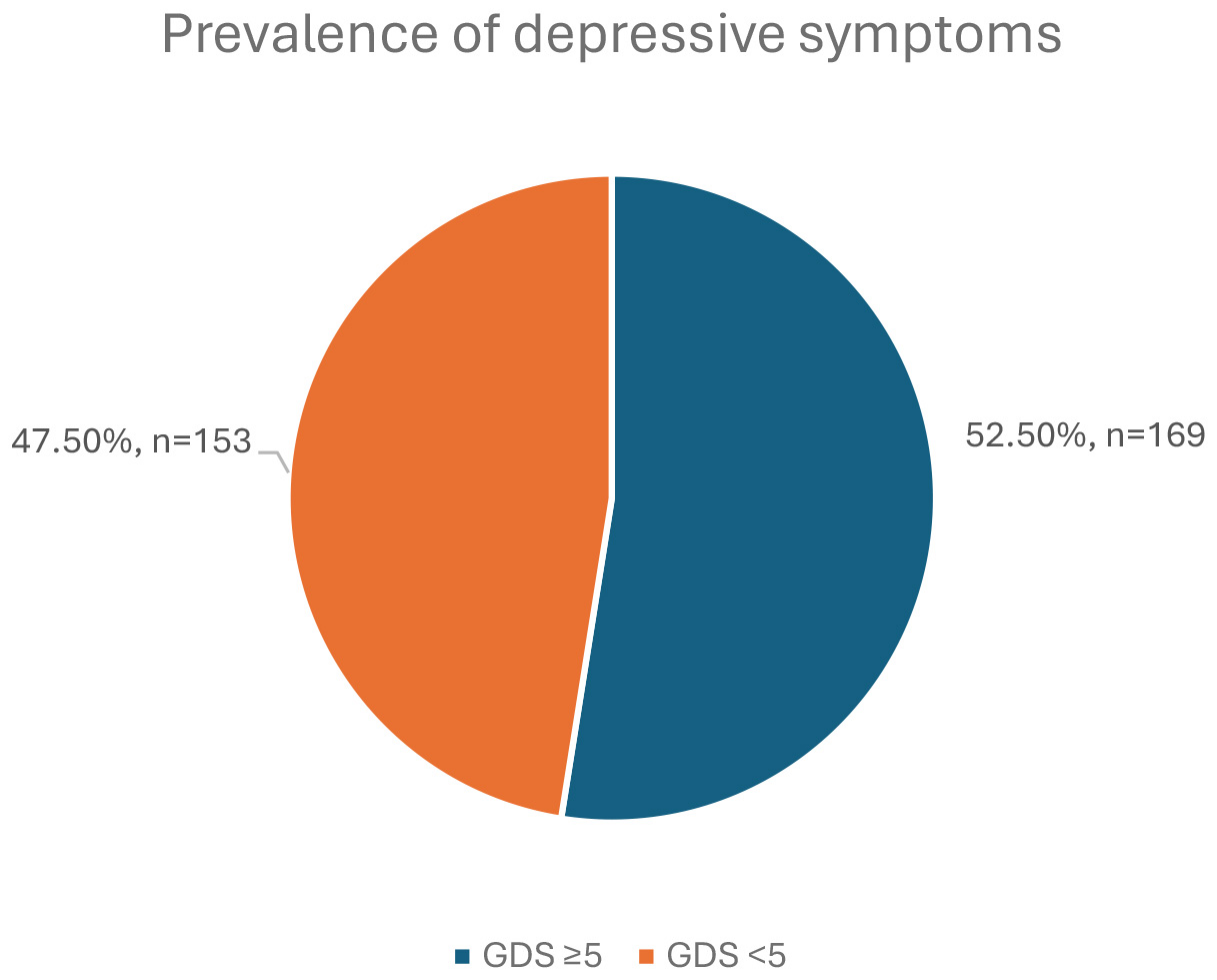
Prevalence of depressive symptoms (GDS-15 ≥5) among older adults in Madinah, Saudi Arabia (*N* = 322).

Using the GDS-15 cutoff of ≥5, the prevalence of depressive symptoms was 52.5% (95% CI: 47.0%−57.9%; *n* = 169 out of 322; Figure 2). Table 2 shows that among the older adults, the median (IQR) GDS-15 score was 5 (6), with a mean (SD) score of 5.45 (3.69), a minimum of 0, and a maximum of 14 out of 15. This indicates that the respondents had, on average, low to moderate depressive symptoms. Additionally, the LSNS-6 had an LSNS (IQR) of 15 (8) and a mean (SD) score of 15.37 (6.08) out of 30 points. This is indicative of the varying degrees of social connectivity among the participants.

**Table 2:** Descriptive statistics of geriatric depression and social network scores among older adults in Madinah.

Factor		Number
GDS total score	Median (IQR)	5 (6)
	Mean (SD)	5.45 (3.69)
	Min. -Max.	0–15
LSNS total score	Median (IQR)	15 (8)
	Mean (SD)	15.37 (6.08)
	Min. -Max.	0–30

Table 3 presents the distribution of depressive symptoms (GDS-15 ≥ 5) across sociodemographic, clinical, and lifestyle factors, along with the statistical significance of each association. Age was significantly associated with depression (*p* = 0.027). Participants aged 71–80 years had the highest prevalence (67.2%), while those

**Table 3:** Factors associated with GDS depressive symptoms among older adults in Madinah, Saudi Arabia.

Factor	Category		GDS ≥5 *n* (%)	GDS < 5 *n* (%)	*P*-value
Age group (years)	60–70		115 (48.5)	122 (51.5)	**0.027**
	71–80		43 (67.2)	21 (32.8)	
	81–90		11 (57.9)	8 (42.1)	
Gender	Female		78 (63.4)	45 (36.6)	**0.002**
	Male		91 (45.7)	108 (54.3)	
Marital status	Single or separate		5 (62.5)	3 (37.5)	**< 0.001** ^*^
	Married		105 (45.3)	127 (54.7)	
	Divorced		6 (50.0)	6 (50.0)	
	Widowed		53 (75.7)	17 (24.3)	
Education level	Illiterate		51 (65.4)	27 (34.6)	**< 0.001**
	Primary to secondary school		72 (59.5)	49 (40.5)	
	Bachelor's degree		37 (35.2)	68 (64.8)	
	Postgraduate		9 (50.0)	9 (50.0)	
Employment status	Unemployed		67 (72.0)	26 (28.0)	**< 0.001**
	Employee		7 (46.7)	8 (53.3)	
	Retired		95 (44.4)	119 (55.6)	
Monthly income	< 3,000		46 (62.2)	28 (37.8)	**< 0.001**
	3,000–5,999		33 (71.7)	13 (28.3)	
	6,000–9,999		41 (53.2)	36 (46.8)	
	>10,000		49 (39.2)	76 (60.8)	
Living arrangement	Live with their families		94 (44.3)	118 (55.7)	**0.001**
	Lives with other family members		20 (69.0)	9 (31.0)	
	Living with children		25 (65.8)	13 (34.2)	
	Living alone		30 (69.8)	13 (30.2)	
Chronic diseases	Diabetes	No	42 (42.9)	56 (57.1)	**0.022**
		Yes	127 (56.7)	97 (43.3)	
	Hypertension	No	78 (45.9)	92 (54.1)	**0.012**
		Yes	91 (59.9)	61 (40.1)	
	Arthritis	No	121 (48.4)	129 (51.6)	**0.006**
		Yes	48 (66.7)	24 (33.3)	
	Respiratory diseases	No	143 (50.0)	143 (50.0)	**0.012**
	Yes	26 (72.2)	10 (27.8)	
Heart disease	No	128 (47.9)	139 (52.1)	**< 0.001**
	Yes	41 (74.5)	14 (25.5)	
Other	No	148 (51.2)	141 (48.8)	0.176
	Yes	21 (63.6)	12 (36.4)	
Nothing	No	165 (55.7)	131 (44.3)	**< 0.001**
	Yes	4 (15.4)	22 (84.6)	
How many prescription medications do you take daily?	None		22 (38.6)	35 (61.4)	**0.025**
	1–2		38 (48.7)	40 (51.3)	
	3 or more		109 (58.3)	78 (41.7)	
How would you rate your overall health?	Excellent		16 (23.2)	53 (76.8)	**< 0.001**
	Very good		49 (40.8)	71 (59.2)	
	Good		72 (75.0)	24 (25.0)	
	Fair to poor		32 (86.5)	5 (13.5)	
How often do you exercise or engage in physical activity?	Daily		5 (20.0)	20 (80.0)	**< 0.001**
	3–5 times a week		11 (36.7)	19 (63.3)	
	1–2 times a week		29 (41.4)	41 (58.6)	
	Rarely		73 (55.7)	58 (44.3)	
	Never		51 (77.3)	15 (22.7)	
Do you have any difficulties with activities of daily living?	No difficulties		75 (38.7)	119 (61.3)	**< 0.001** ^*^
	Some difficulties		60 (67.4)	29 (32.6)	
	Many difficulties		27 (84.4)	5 (15.6)	
	Unable to perform without help		7 (100.0)	0 (0.0)	

Chi-Square Test, ^*^Fisher Exact Test. Bold values indicate statistical significance at the *p* < 0.05 level.

aged 60–70 years had the lowest (48.5%). Gender showed a strong association (*p* = 0.002), where depression was more common among females (63.4%) than males (45.7%). Marital status was highly significant (*p* < 0.001). Widowed participants had the highest depression rate (75.7%).

Education level was similarly significant (*p* < 0.001), with depression prevalence decreasing from illiterate (65.4%) and primary-secondary (59.5%) to bachelor's degree (35.2%), though postgraduate participants showed a rate of 50.0%. Employment status (*p* < 0.001) revealed that unemployed individuals had the highest depression rate (72.0%), compared to employees (46.7%) and retirees (44.4%).

Monthly income (*p* < 0.001) demonstrated a gradient, with the lowest income group (< 3000 SAR) had 62.2% depression, the 3000–5999 SAR group the highest (71.7%), and the highest income group (>10000 SAR) the lowest (39.2%). Living arrangement (*p* = 0.001) indicated that those living alone or with other family members had higher depression rates (69.8 and 69.0%, respectively) than those living with their own families (44.3%).

Among chronic diseases, all except “other chronic disease” were significantly associated with depression. Participants with heart disease had the highest prevalence (74.5%, *p* < 0.001), followed by respiratory disease (72.2%, *p* = 0.012), arthritis (66.7%, *p* = 0.006), hypertension (59.9%, *p* = 0.012), and diabetes (56.7%, *p* = 0.022); those reporting no chronic disease had the lowest rate (15.4%, *p* < 0.001). “Other chronic disease” was not significant (63.6 vs. 51.2%, *p* = 0.176).

Number of daily prescription medications showed a significant trend (*p* = 0.025), with depression prevalence increased from 38.6% (none) to 48.7% (1, 2) to 58.3% (≥3). Self-rated health demonstrated a strong gradient (*p* < 0.001), rising from 23.2% (excellent) to 40.8% (very good), 75.0% (good), and 86.5% (fair/poor). Physical activity frequency was also highly significant (*p* < 0.001): those who never exercised had the highest depression rate (77.3%) while daily exercisers had the lowest (20.0%). Finally, difficulties with activities of daily living (ADLs) showed the strongest association (*p* < 0.001, Fisher's exact), with depression prevalence increasing progressively from 38.7 (no difficulties) to 67.4% (some difficulties), 84.4% (many difficulties), and 100% (unable without help).

Table 4 presents the analysis of social connectedness using the LSNS-6, which revealed several significant associations. Social network scores had a median (IQR) score of 16 (8) in the younger participant age group, compared with other age groups (*p* = 0.006), and between male and female participants, 16 (8) compared to females (*p* = 0.003). Education was significant, with participants who completed primary (*p* = 0.010) or secondary school reporting 16 (8) years higher education than those who were illiterate or had a postgraduate degree. The living environment was also significant, with people living alone scoring the lowest at 12 (8) compared with individuals in joint or group accommodation (*p* = 0.009). Hypertension (*p* = 0.004), arthritis (*p* = 0.034), and heart disease (*p* = 0.019) were associated with lower LSNS scores. In contrast, those reporting other conditions paradoxically had higher scores than those not reporting any other conditions (*p* = 0.012). Polypharmacy was apparent, with participants taking three or more medications daily scoring lower 14 (6) than those on fewer or none (*p* = 0.005). Self-rated health showed a clear association, with the highest median (IQR) score of 19 (6) for excellent and the lowest scores for fair to poor (*p* < 0.001). Similarly, regular physical activity was positively correlated with the level of social connectedness, with daily exercisers having the strongest networks, as indicated by a median (IQR) score of 19 (9) (*p* < 0.001). Lastly, functional independence was also an important determinant, and those not experiencing Activity Daily Live problems had significantly higher LSNS median (IQR) scores of 16 (7) compared to their counterparts who experienced many limitations or depended on help (*p* < 0.001).

**Table 4:** Factors associated with LSNS total score among older adults in Madinah, Saudi Arabia.

Factor	Category		LSNS total score median (IQR)	*P*-value
Age group (years)	60–70		16 (8)	**0.006** ^**^
	71–80		13 (8)	
	81–90		14 (8)	
Gender	Female		14 (9)	**0.003** ^*^
	Male		16 (8)	
Marital status	Single or separate		17.5 (10)	0.797^**^
	Married		16 (8)	
	Divorced		14 (6)	
	Widowed		12.5 (9)	
Education level	Illiterate		14 (8)	**0.010** ^**^
	Primary to secondary school		16 (8)	
	Bachelor's degree		15 (8)	
	Postgraduate		13 (11)	
Employment status	Unemployed		14 (10)	0.196^**^
	Employee		16 (9)	
	Retired		15 (8)	
Monthly income	< 3,000		13.5 (8)	0.052^**^
	3,000–5,999		16 (2)	
	6,000–9,999		16 (7)	
	>10,000		15 (7)	
Living arrangement	Live with their families		15 (7)	**0.009** ^**^
Lives with other family members		15 (10)	
Living with children		15.5 (9)	
Living alone		12 (8)	
Chronic diseases	Diabetes	No	16 (10)	0.107^*^
		Yes	15 (8)	
	Hypertension	No	15.5 (7)	**0.004** ^*^
		Yes	14 (8)	
	Arthritis	No	15 (8)	**0.034** ^*^
		Yes	14 (8)	
	Respiratory diseases	No	15 (7)	0.201^*^
	Yes	14 (10)	
Heart disease	No	15 (8)	**0.019** ^*^
	Yes	14 (8)	
Other	No	15 (8)	**0.012** ^*^
	Yes	19 (8)	
Nothing	No	15 (8)	0.402^*^
	Yes	15.5 (11)	
How many prescription medications do you take daily?	None		16 (11)	**0.005** ^**^
	1–2		18 (9)	
	3 or more		14 (6)	
How would you rate your overall health?	Excellent		19 (6)	**< 0.001** ^**^
	Very good		16 (7)	
	Good		13 (8)	
	Fair to poor		12 (8)	
How often do you exercise or engage in physical activity?	Daily		19 (9)	**< 0.001** ^**^
	3–5 times a week		18.5 (6)	
	1–2 times a week		15 (7)	
	Rarely		13 (9)	
	Never		15 (8)	
Do you have any difficulties with activities of daily living?	No difficulties		16 (7)	**< 0.001** ^**^
	Some difficulties		15 (9)	
	Many difficulties		12 (6)	
	Unable to perform without help		12 (7)	

^*^Mann-Whitney Test.

^**^Kruskal-Wallis Test. Bold values indicate statistical significance at the *p* < 0.05 level.

Table 5 presents the results of multivariable logistic regression analysis identifying independent predictors of depressive symptoms (GDS-15 ≥ 5). Unemployment emerged as a strong independent risk factor: unemployed participants had nearly eight-fold higher odds of depression compared to retirees (adjusted OR = 7.66, 95% CI: 2.12–27.66, *p* = 0.002). Notably, having no chronic disease was associated with a ten-fold increase in depression odds relative to having at least one chronic condition (OR = 10.62, 95% CI: 1.91–58.89, *p* = 0.007).

**Table 5:** Multivariable logistic regression analysis of factors associated with depressive symptoms (GDS-15 ≥ 5) among older adults in Madinah (*N* = 320).

Factor	Category		Adjusted OR	95% CI	*p*-value
Age group (ref: 81–90 years)	60–70		2.89	0.53–15.79	0.221
	71–80		3.55	0.66–19.01	0.139
Gender (ref: male)	Female		0.67	0.25–1.79	0.420
Marital status (ref: widowed)	Single or separate		5.67	0.74–43.75	0.096
	Married		1.63	0.42–6.35	0.481
	Divorced		1.71	0.29–10.25	0.556
Education level (ref: postgraduate)	Illiterate		2.17	0.41–11.58	0.364
	Primary to secondary school		2.54	0.56–11.47	0.225
	Bachelor's degree		1.12	0.28–4.59	0.871
Employment status (ref: retired)	Unemployed		7.66	2.12–27.66	**0.002**
	Employee		3.07	0.77–12.35	0.114
Monthly income (ref: >10,000 SAR)	< 3,000		0.34	0.09–1.38	0.131
	3,000–5,999		1.54	0.50–4.75	0.452
	6,000–9,999		1.08	0.49–2.37	0.847
Living arrangement (ref: alone)	Live with their families		2.40	0.51–11.32	0.270
	Lives with other family members		1.84	0.45–7.56	0.395
	Living with children		3.99	0.90–17.73	0.069
Chronic diseases	Diabetes (ref: no)	Yes	0.53	0.23–1.22	0.134
	Hypertension (ref: no)	Yes	1.04	0.53–2.03	0.905
	Arthritis (ref: no)	Yes	0.99	0.43–2.31	0.988
	Respiratory diseases (ref: no)	Yes	1.06	0.39–2.90	0.910
	Heart disease (ref: no)	Yes	0.78	0.32–1.95	0.602
	No chronic disease (ref: has ≥1 disease)	Yes	10.62	1.91–58.89	**0.007**
Number of medications (ref: 3 or more)	None		3.93	1.20–12.90	**0.024**
	1–2		0.90	0.41–1.99	0.794
Self-rated health (ref: fair/poor)	Excellent		0.18	0.04–0.83	**0.028**
	Very good		0.26	0.06–1.08	0.064
	Good		0.97	0.25–3.75	0.963
Physical activity frequency (ref: never)	Daily		0.15	0.04–0.62	**0.009**
	3–5 times a week		0.19	0.06–0.68	**0.011**
	1–2 times a week		0.17	0.06–0.47	**0.001**
	Rarely		0.25	0.10–0.60	**0.002**

Bold values indicate statistical significance at the *p* < 0.05 level.

Regarding number of medications, taking no medications (compared to three or more) was associated with higher depression odds (OR = 3.93, 95% CI: 1.20–12.90, *p* = 0.024), while the 1-2 medications category was not significant. Self-rated health showed a protective gradient: excellent health (vs. fair/poor) was associated with 82% lower odds of depression (OR = 0.18, 95% CI: 0.04–0.83, *p* = 0.028), whereas very good and good health did not reach statistical significance. Physical activity was strongly protective across all frequencies: compared to never exercising, daily exercise (OR = 0.15, *p* = 0.009), 3-5 times/week (OR = 0.19, *p* = 0.011), 1-2 times/week (OR = 0.17, *p* = 0.001), and even rare exercise (OR = 0.25, *p* = 0.002) all significantly reduced depression odds.

Table 6 presents the results of multivariable linear regression analysis examining factors independently associated with social connectedness, as measured by the LSNS-6 total score. The model explained 15.2% of the variance in LSNS-6 scores (*R*^2^ = 0.152) and was statistically significant (*p* < 0.001). After adjusting for all covariates, having an “other” chronic disease was significantly associated with higher LSNS-6 scores (*B* = 3.357, 95% CI: 1.12–5.60, *p* = 0.003). In contrast, poorer self-rated health was significantly associated with lower LSNS-6 scores (*B* = −1.703, 95% CI: −2.65 to −0.76, *p* < 0.001). None of the other variables reached statistical significance.

**Table 6:** Linear regression analysis of factors associated with social connectedness (LSNS 6 total score) among older adults in Madinah (*N* = 320).

Factor	Unstandardized *B*	95% CI	*p*-value
Age group (ref: 81–90 years)	−0.172	[−1.49–1.15]	0.798
Gender (ref: male)	0.224	[−1.80–2.25]	0.828
Marital status (ref: widowed)	−0.007	[−1.23–1.22]	0.991
Education level (ref: postgraduate)	0.163	[−0.86–1.18]	0.753
Employment status (ref: retired)	−0.050	[−1.16–1.06]	0.929
Monthly income (ref: >10,000 SAR)	0.112	[−0.71–0.94]	0.789
Living arrangement (ref: alone)	−0.160	[−0.99–0.67]	0.706
Diabetes (ref: no)	−0.241	[−2.04–1.56]	0.793
Hypertension (ref: no)	−0.110	[−1.67–1.45]	0.890
Arthritis (ref: no)	0.534	[−1.23–2.30]	0.552
Respiratory diseases (ref: no)	0.069	[−2.04 – 2.18]	0.949
Heart disease (ref: no)	0.502	[−1.41–2.41]	0.606
Other (ref: no)	3.357	[1.12–5.60]	**0.003**
Number of medications (ref: 3 or more)	−0.421	[−1.64–0.80]	0.497
Self-rated health (ref: fair/poor)	−1.703	[−2.65 – −0.76]	**< 0.001**
Physical activity frequency (ref: never)	−0.381	[−1.02–0.26]	0.241
Daily living activities difficulties (ref: Unable to perform without help)	−0.233	[−1.37–0.90]	0.686

Bold values indicate statistical significance at the *p* < 0.05 level.

## Discussion

Depression is a major public health problem throughout the world, and older adults are at greater risk due to changes that occur in their mind, body, and social life. However, the mental health of the older adults is an increasingly significant concern with the growing number of this population subgroup, as in Saudi Arabia, due to rapid demographic transition, and for similar countries. By studying depression in older adults, they could look for ways to create better health care plans and support specifically for them (21). This study investigated the prevalence and correlates of depression among older adults in Madinah, Saudi Arabia. Using GDS-15 and LSNS-6 questionnaires. The mean (SD) GDS score was 5.45 (3.69); the median (IQR) was 5 (6), suggesting that a large share of participants had depressive symptoms.

The prevalence of depressive symptoms (GDS-15 ≥5) was 52.5%, which is substantially higher than the 39% reported in a Saudi study 3 decades ago (15) and higher than recent estimates from other Gulf countries (e.g., 28% in Oman, 31% in Qatar). This difference may reflect true increases in geriatric depression due to rapid social change, or variations in screening methods and sampling (22). Importantly, our multivariable analysis identified unemployment, having no chronic disease, taking no medications, poor self-rated health, and physical inactivity as independent interpreters to depression, while many sociodemographic factors lost significance after adjustment.

This study found that there was an age-related increase in the depression scores: participants aged between 81 and 90 years reached a higher median GDS score of 8, which was statistically significant compared to the younger ages (*p* = 0.003). This gradient is also in line with other Saudi studies, which found higher rates of depression among the older age groups (15). Likewise, another study in 2006 underlined that depression is largely influenced by advancing age as a factor of increasing morbidity, frailty, and loneliness. These results indicated a higher depression risk and lower social networks (23). Furthermore, this is consistent with international trends in which the risk of vulnerability increases with age, in part because it is a summation of losses arising from retirement or bereavement (24). There were similar age-related increases in prevalence in a study from Taif, Saudi Arabia, with the largest increase among those aged >75 years who reported less independence (25).

After multivariable adjustment, age was no longer significantly associated with depression (*p* > 0.05), suggesting that the bivariate age effect was confounded by other factors such as physical activity, self-rated health, and chronic disease.

This study found that females (38.2% of the sample) reported higher depression scores than did males (*p* < 0.001). This aligns with another study conducted in 2023, which showed that women in Saudi Arabia report higher levels of depression (21). The evidence provided globally suggests that gender is a ubiquitous risk factor for late-life psychopathology (26). This discrepancy could be attributed to biological vulnerability, longer life span, and increased exposure to widowhood, as well as the burden of care (27). Gender, such as Female sex, is a recognized risk factor around the world, with pathways associated in some cases with hormonal change, caregiving roles, and social inequalities (28). Saudi-specific (Makkah and Riyadh) literature confirms this, wherein older adults females report 10%−20% higher prevalence of depression attributed mostly to cultural constraints restricting ways of sharing mobility and support (18).

In the multivariable model, gender was not independently associated with depression (*p* = 0.420), indicating that the bivariate gender difference was explained by other factors such as marital status, income, and physical activity (29).

Additionally, this study shows that the Depression score of illiterates (24.2%) was significantly higher than that of those having a bachelor's degree (*p* < 0.001). Similarly, participants with a lower income had significantly higher depression scores than those with a higher income (*p* < 0.001). These results are consistent with another study, which reported that low education and financial difficulties were the main predictors for depression among Saudi adults (21). That means Education is the raw material for cognitive reserve and adaptive coping, as demonstrated in international reviews with low literacy associated with depressed a higher risk of depression (30). Older Saudis with little education resemble this profile, exacerbating economic disadvantage (31). Education and income lost significance after adjustment, suggesting that their effects are mediated by employment status and self-rated health.

This study demonstrates that there are strong correlations between chronic diseases and depression. The participants with hypertension and arthritis had a higher median GDS score, which was significantly higher than those without these illnesses (*p* < 0.001). Diabetes (69.6%) and depression scores were also the same in individuals who had higher levels of diabetes patients (*p* = 0.012). These findings are analogous to another study conducted in 2025, which found that frailty and chronic illness greatly raise the risk of depression in Saudi older adults (32). Additionally, polypharmacy burden was reflected by the fact that those on polypharmacy (58.1% were using more than three drugs daily) had higher depression scores when compared to patients taking none (*p* = 0.002), which was similar to Saudi studies noted in the hospitalized patients (33). Additionally, the proportion of older adults living alone (13.4%) had the highest depression symptom scores (*p* < 0.001) compared to those living with family. This finding aligns with the protective effect of family support in Saudi society, as reported in a 2023 study (34). LSNS also confirmed that widowed patients and those living alone scored significantly fewer social network points than married patients (*p* = 0.009), a finding consistent with another study (15).

In the multivariable model, individual chronic diseases (diabetes, hypertension, arthritis, respiratory disease, and heart disease) were not significant indicators of depression. However, the unexpected finding that having no chronic disease was associated with ten-fold higher depression odds (OR = 10.62, 95% CI: 1.91–58.89, *p* = 0.007).

Comparison with regional literature reveals both similarities and differences. Unemployment was consistently identified as a significant contributor to depression in Egypt (35) and a significant correlate in Jordan (36). The protective effect of physical activity is consistent across the Gulf, with a recent Bahrain case-control study confirming that low physical activity is evident in patients with depression (37). However, the “no chronic disease” paradox has not been reported in other Arab studies, suggesting it may be a chance finding or specific to Madinah's older adults population.

Regarding social connectedness (LSNS-6), the positive association between “other” chronic disease and higher LSNS-6 scores (*B* = 3.357, 95% CI: 1.12–5.60, *p* = 0.003) is unexpected. One interpretation is that minor or visible conditions may elicit greater sympathy and support from family and friends, strengthening social ties. Alternatively, this category may include participants with higher socioeconomic status who have both better access to diagnosis of minor ailments and larger social networks. Poorer self-rated health was associated with lower LSNS-6 scores (*B* = −1.703, *p* < 0.001), reinforcing that perceived health status is a strong correlate of social isolation independent of objective disease burden.

Overall, from this study, we realized that depression among older people in Madinah depends on a complex interaction of demographic, socioeconomic, medical, and psychosocial factors. The findings are supported by Saudi and international literature and have explored the importance of integrated, culturally sensitive interventions that target not only medical comorbidities but also social support, physical activity, and functional independence.

## Limitations

As this study's cross-sectional design does not allow for causal relationships between depression and related factors to be determined, all identified associations should be interpreted as correlations only, not as causal effects. However, the study serves as the baseline for a planned longitudinal follow-up of the same cohort, which will help establish temporality. Additionally, the study used self-reported instruments such as GDS and LSNS; thus, recall bias and an underestimation of mood-related problems due to social stigma might be potential issues.

## Conclusion

In bivariate analyses, depression was more common among women, the widowed, older age groups, those with lower education and income, and individuals living alone. However, after multivariable adjustment, the independent correlates of depression were unemployment (OR = 7.66), having no chronic disease (OR = 10.62), taking no medications (OR = 3.93), poor self-rated health (OR = 0.18 for excellent health), and physical inactivity (daily exercise OR = 0.15). The unexpected finding that the absence of chronic disease was associated with higher depression odds warrants further investigation and may reflect reverse causality, health anxiety, or cultural factors related to illness-related social support. Regarding social connectedness, poorer self-rated health was associated with lower LSNS-6 scores (greater social isolation), while having an “other” chronic disease was unexpectedly linked to better social networks.

Given the cross-sectional design, causality cannot be inferred; planned longitudinal follow-up will help establish temporal relationships. Nonetheless, these findings underscore the urgent need for integrated, culturally tailored interventions in Saudi Arabia that promote physical activity, address unemployment and financial insecurity, improve mental health literacy, and reduce stigma—particularly among older adults who perceive themselves as healthy but may be at unrecognized risk for depression. Early detection through routine screening in primary care and community settings should be prioritized to improve quality of life in the aging Saudi population.

## Data Availability

The raw data supporting the conclusions of this article will be made available by the authors, without undue reservation.
